# The Texture of Camel Milk Cheese: Effects of Milk Composition, Coagulants, and Processing Conditions

**DOI:** 10.3389/fnut.2022.868320

**Published:** 2022-04-19

**Authors:** Mustapha Mbye, Mutamed Ayyash, Basim Abu-Jdayil, Afaf Kamal-Eldin

**Affiliations:** ^1^Department of Food Science, United Arab Emirates University, Al-Ain, United Arab Emirates; ^2^Department of Petroleum & Chemical Engineering, United Arab Emirates University, Al-Ain, United Arab Emirates

**Keywords:** camel milk cheese, pasteurization, high pressure processing, coagulants, bovine milk cheese

## Abstract

Numerous people in African, Middle Asian, Middle Eastern, and Gulf Cooperation Council (GCC) countries highly value camel milk (CM) as it plays a vital role in their diet. The protein composition of CM as well as the structure of its casein micelles differs significantly from bovine milk (BM). Cheeses made from CM have a weak curd and soft texture compared to those made from BM. This review article presents and discusses the effect of milk protein composition, processing conditions (pasteurization and high-pressure treatment), and coagulants (camel chymosin, organic acids, plant proteases) on the quality of CM cheeses. CM cheese's weak texture is due to compositional characteristics of the milk, including low κ-casein-to-β-casein ratio (≈0.05 in CM vs. ≈0.33 in BM), large micelle size, different whey protein components, and higher proteolytic activity than BM. CM cheese texture can be improved by preheating the milk at low temperatures or by high pressure. Supplementing CM with calcium has shown inconsistent results on cheese texture, which may be due to interactions with other processing conditions. Despite their structure, CM cheeses are generally well liked in sensory studies.

## Introduction

The world's camel population is approximately 35 million with Dromedary one-humped camels (*Camelus dromedarius*) representing around 95% and Bactrian camels (*Camelus bactrianus*) constituting the rest (1). Camels are able to survive harsh, hot, and dry climatic conditions and produce milk for a more extended period than any other milch animal under the same arid conditions with despite a low milk yield. The global production of camel milk (CM) is increasing by about 2.45% yearly (2) for at least three reasons: (i) contribution to food security in marginal environments, (ii) new market opportunities due to unique health properties, and (iii) development of camel dairy industries, which could be profitable for settled producers (3). In recent years, the health benefits of CM and its products have attracted much attention to the possibilities of its use as an alternative to bovine milk (BM) (4, 5). Several nutritional and therapeutic effects have been reported such as anti-diabetic (6–8), though large-scale clinical studies still are lacking. CM has also been promoted as a viable alternative to BM for children who are allergic to cow's milk (9). The effect of CM consumption on autism disorders was examined by evaluating 65 children with autism (10). The study demonstrated that children with Autism Spectrum Disorder (ASD) showed significant improvements after 2 weeks of camel milk consumption compared to the placebo group. A study on rats showed CM-treated rats had reduced Bcl2 mRNA levels in their tumor tissues compared to the control group (12). CM has also been proposed to have antimicrobial activity (13), and CM fermented with *Lactobacillus helveticus* has been shown to have inhibitory effect against angiotensin I-converting enzyme which is known to cause lowering of blood pressure (14).

**Figure 1:**
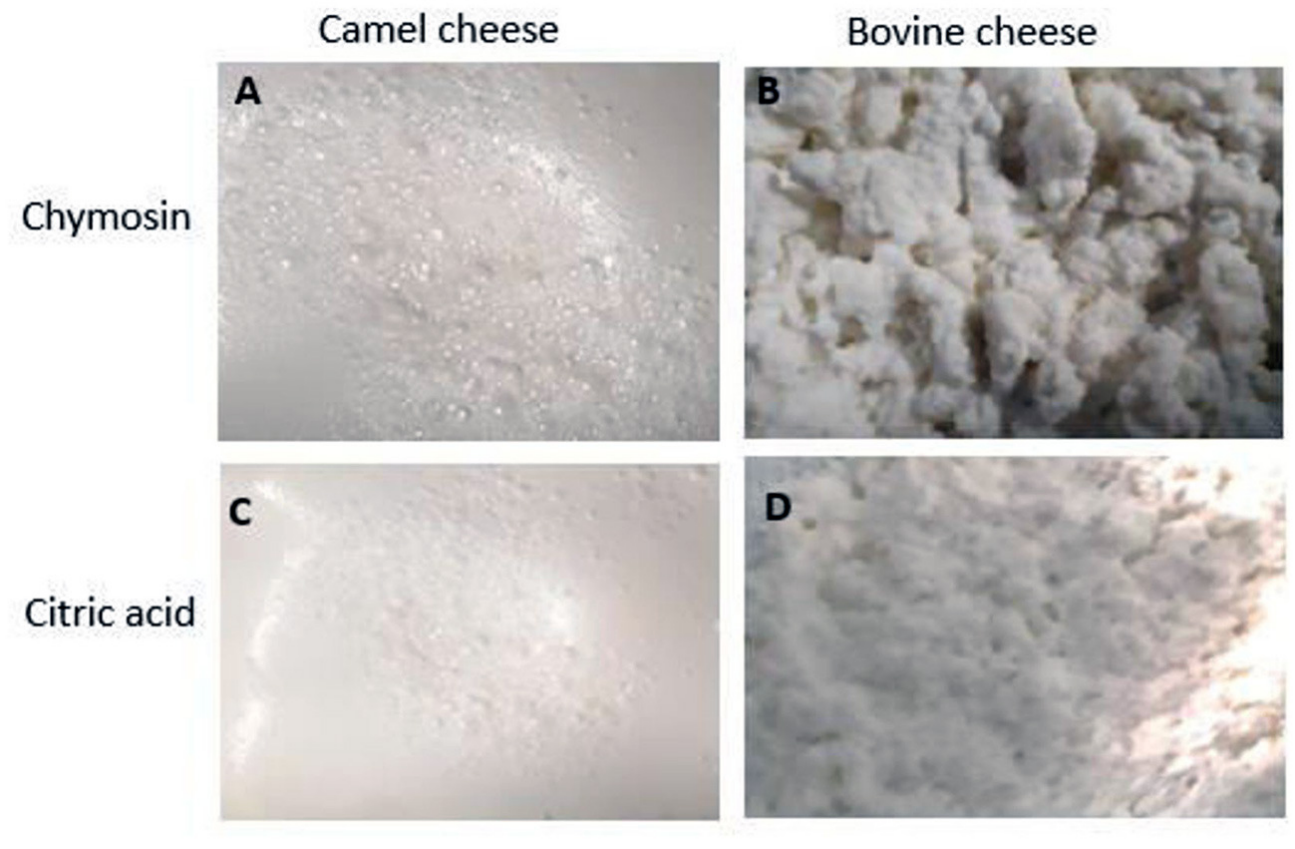
Photographs of camel and bovine milk cheeses produced by using chymosin (50 IMCU/L milk) or 30%citric acid. **(A)** Camel milk cheese made with chymosin, **(B)** Bovine milk cheese made with chymosin, **(C)** Camel milk cheese made with citric acid, and **(D)** Bovine milk cheese made with citric acid.

To increase its shelf life and market opportunities, CM must be processed into products that can be stored for extended periods and easily transported, such as cheese, yogurts, and milk powders. However, previous studies have reported that the transformation of CM into cheese is challenging and the produced cheese is always softer than cheese produced from BM (15–17). Figure 1 shows the difference between CM and BM fresh model cheeses produced by chymosin or citric acid precipitation. CM cheeses are generally soft and smooth compared to those produced from BM and the time needed for their coagulation using recombinant camel chymosin has been shown to be 2–4 times longer than that needed for BM (11, 18). CM cheeses have been shown (11) to exhibit higher acidity and lower hardness than those of BM (Table 1). Despite these differences, consumers evaluate CM cheeses positively (19), suggesting that CM cheeses can be produced and commercialized as special quality cheeses with possible health advantages (11). In this review article, we discuss the different properties of cheeses made from CM in comparison to BM and how they are affected by milk composition, processing conditions, and coagulation agents.

**Table 1:** Physicochemical, yield, hardness, and rheological properties and moisture content of the camel and bovine cheeses*.

**Parameter**	**Chymosin**		**Citric acid**	
	**CM**	**BM**	**CM**	**BM**
Yield (%) (g cheese/100 g milk)	12.3 ± 1.2^ab^	9.6 ± 1.6^b^	13.6 ± 1.6^a^	11.0 ± 1.5^ab^
Moisture content (g/100 g cheese)	66 ± 3.6^bc^	48 ± 3.6^d^	67 ± 0.4^ab^	62 ± 0.2^c^
pH	4.7 ± 0.01^ab^	4.9 ± 0.12^a^	4.4 ± 0.1^bc^	4.3 ± 0.1^c^
Acidity (%)	1.5 ± 0.3^cd^	1.1 ± 0.2^d^	2.4 ± 0.1^a^	1.9 ± 0.2^bc^
Hardness (g)	422 ± 49^c^	4,612 ± 451^a^	342 ± 11^c^	3,331 ± 199^b^
Complex Viscosity (Pa.s)	377 ± 44^c^	2,150 ± 87^a^	329 ± 25^c^	2,051 ± 51^a^
G′, Pa	2,678 ± 445^c^	20,953 ± 577^a^	1,929 ± 209^c^	16,032± 514^b^
G″, Pa	975 ± 75^b^	4,383 ± 390^a^	693 ± 56^b^	4,238 ± 306^a^

* *Means within a row with different alphabetical superscripts are significantly different (P < 0.05, n = 3). Source: Mbye et al. (11)*.

## Effect of Milk Composition on Cheese Quality

The content of major constituents and the protein compositions of CM and BM are presented in Table 2. The overall composition is comparable between the two milks (23) but significant differences are evident in the protein composition (24, 25). Although CM and BM have comparable titrable acidity, CM has a higher buffering capacity (26). The curd production phases (i.e., enzymatic coagulation, curd firming, and syneresis) are all influenced by the milk composition, particularly by concentration and types of caseins, fat, pH, and calcium (27). Milk composition, particularly protein and fat contents and composition, will also significantly affect cheese yield and composition (28).

**Table 2:** Average pH/acidity, content of major constituents, and protein composition of camel and bovine milks*.

**Components**	**Camel milk**	**Bovine milk**
Acidity (%)	0.13	0.11
pH	6.68	6.68
Total solids (%)	12	13
Fat (%)	3.5	3.7
Lactose (%)	4.4	4.8
Ash (%)	0.8	0.7
Total protein (%)	3.1	3.4
**Caseins**
α_s1_-casein (g/l)	5.3 (22%)	9.5 (38%)
α_s2_-casein (g/l)	2.3 (9.6%)	2.5 (10%)
β-casein (g/l)	15.6 (65%)	9.8 (39%)
κ-casein (g/l)	0.8 (3.3%)	3.3 (13%)
Total casein content as % of the proteins	2.4/ 3.1 (77%)	2.51/3.4 (74%)
**Whey proteins**
β-Lactoglobulin (g/l)	Not present	3.3 (53.6%)
α-Lactalbumin (g/l)	2.3 (27%)	1.1 (20.1%)
Serum albumin (g/l)	2.2 (26%)	0.35 (6.2%)
Whey acidic protein (g/l)	0.16 (1.8%)	Not present
Lactoferrin (g/l)	0.18 (2.0%)	0.10 (1.7%)
Immunoglobulins IgA, IgG, IgM (g/l)	1.5 (18%)	0.30 (5.3%)

*Sources: Al Kanhal (20); Hailu et al. (21); Li et al. (22)*.

The milk's casein content affects the coagulation and gel forming rates, which increase with increased casein concentration (29). Casein, the main milk component affecting cheese quality, was reported to represent ~77% of total CM proteins which is similar to BM (Table 2). The casein composition (g/l) of CM vs. BM is as follow: α-s1 (5.3 vs. 9.5), α-s2 (2.3 vs. 2.5), ß (15.6 vs. 9.8), and κ (0.8 vs. 3.3), respectively (21) (Table 2). The relative concentrations of αs1-, αs2-, β-, and κ- caseins are ~22:9.5: 65:3. Five in CM (24) compared to 40:10:40:10 in BM (30). Low κ-casein contents in CM have been associated with poor milk curdling properties and low cheese yield (31). The casein micelles of CM are also larger (260–300 nm in diameter) than those of BM (100–140 nm in diameter) (32, 33). For BM, large casein micelles with reduced surface area has been associated with increased rennet coagulation time, reduced cheese curd firmness, and lower overall cheese quality (34). For CM, the low level of k-casein coupled with the large micelle size has been considered as the main factor responsible for the weak coagulation of CM (17, 31, 35–37).

The whey proteins represent about 23% of the total proteins of CM (20) similar to the ~20% in BM (38). A noteworthy difference between CM and BM whey proteins is that CM lacks β-lactoglobulin (39), which has important implications on milk functional properties mainly through its heat-induced association with κ-casein (40). In comparison, α-lactalbumin is the major whey protein representing about 50% of CM whey proteins compared to 25% of BM whey proteins (39, 41). The other whey proteins in CM include *inter alias* serum albumin, lactoferrin, acidic whey protein, glycosylation-dependent cell adhesion molecule 1, peptidoglycan recognition protein, lactoperoxidase, and immunoglobulins (21, 25, 42). Some of these proteins, e.g., lysozyme, lactoferrin, and lactoperoxidase have antimicrobial properties and have been speculated to slow bacterial growth in CM (43, 44). For example, a maximum acidification rate of 12 h and lag phase of 5 h was observed in CM fermented with lactic acid bacteria compared to 6 and 1 h in BM, respectively (26). However, Berhe et al. (45) investigated the growth of eight commercial starter cultures in CM and BM and concluded that the cultures were not inhibited by CM but that the growth rate was restricted due to a more limited rate of proteolysis.

Cheese quality and yield are also affected by the contents and composition of the fat in milk (27). CM fat is packed in smaller fat globules (3.2–5.6 μm diameter) compared with BM fat globules (4.3–8.4 μm diameter) (46). The smaller fat globules of CM may contribute to its soft cheese texture and additionally provides higher *in vitro* digestibility than BM (47). It is essential to standardize milk based on the protein to fat ratio prior to cheese manufacturing (48). For example, the proportion of protein to fat should be 0.84–1.02 for Cheddar cheese according to the specifications of the Irish cheese industry with protein contents ranging 2.99–3.59% and fat contents ranging 3.3–4.2% (48). The mean values of CM protein and fat (3.1 and 3.5%, respectively) fall within this range (20).

Table 3 presents an overview of the studies performed on preparation of CM cheese as affected by milk composition, coagulants, and processing conditions. Increasing total milk total solids and changing protein composition, e.g., by adding milks of other animals (49, 51, 52), milk powders (54, 55, 77), sweet potato powder (53), or by ultrafiltration (56, 57), have all been applied to improve the cheese quality. Mixing CM with milks from other animals alters the content of total protein, fat, as well as the casein composition of the mixed milk and the resultant cheese. For example, combining CM with buffalo milk was found to increase the total solids, fat, ash, and protein contents in soft cheeses and to enhance the organoleptic properties of the cheeses (49, 50, 78). A study by Shahein et al. (50) documented that mixing CM with buffalo milk reduced the rennet coagulation time and the loss of total solids into whey compared to using only CM due to improved curd firmness. Saadi et al. (52) reported that mixing 50% CM with 50%, sheep milk improved cheese texture, fat and protein. Habtegebriel and Emire (54) showed that camel cheese yield increased by 14.9% by adjusting the fat level to 1.8%. Compared to bovine milk, CM cheeses are softer but are, nevertheless, liked by the consumers (11, 19).

**Table 3:** The effect of milk components, coagulants, and processing condition on the quality of CM cheese.

**References**	**Objective**	**Processing method**	**Key findings**
**Comparing CM with other milks and effect of milk composition**			
Inayat et al. (49)	To compare the quality of unripened CM cheese with buffalo milk cheese	Pasteurization (90°C for 10 min), cooling (40°C), addition of rennet, coagulation (5 h)	Buffalo milk cheese had a better yield and sensory score than CM cheese
Shahein et al. (50)	To evaluate the effect of mixing CM and bovine milk on soft cheese yield and curd properties	Cheese made from CM mixed with buffalo milk (90:10, 80:20, 70:30, and 60:40%) by heating (37°C) with 0.04 % calcium chloride. Cheeses stored in plastic boxes of polystyrene in the whey syneresis for 60 days at 5°C	Mixing CM with buffalo milk increased cheese yield, hardness, total solids, fat, ash, protein contents, and decreased weight loss and organoleptic properties during pickling
Derar and El Zubeir (51)	To evaluate the properties and sensory quality of fresh soft cheeses made from camel and sheep milks mixtures	Cheese made from mixture of camel & sheep's milks (25, 50, and 75%), CaCl_2_, Camifloc enzyme	Mixing camel and sheep milks at 50 and 75% levels reduced the coagulation time and improved the cheese texture
Saadi et al. (52)	To investigation the chemical composition of cheese made from CM or a mixture of CM and sheep milk	Pasteurization (71°C, 30 s), trypsin enzyme (0.5 g), CaCl_2_ (0.5 g), in 5 kg mixtures of CM and Sheep milk of milk: (T1) 100% CM, (T2) 75% CM, (T3) 25% CM, (T4) 50% CM, and (T5) 0% CM	Solids, fat, and protein percentages increased with increased sheep milk percentage and cheese quality improved
Elnemr et al. (53)	To evaluate the effect of supplementing CM with a milky component (BMR) and sweet potato powder (SPP) on cheese quality	CM supplementation with BMR (20 or 30%) and SPP (1, 2, or 3%), heating (65°C, 30 min), cooling to (42°C), addition of calcium chloride (0.04%) and sodium chloride (3%), and 1% yogurt culture (42°C, for 30 min), and bovine pepsin (4 mg/100 g), drainage (24 h), packing in plastic containers (in 5% brine solution, 4 weeks), and refrigeration at 5°C	Fortification of CM with BMR and SPP reduced the pepsin coagulation time, whey syneresis, and the pH value, and improved the physic-chemical properties of brined cheese
Habtegebriel and Emire (54)	To evaluate the effect of total solids, fat content, and amount of coagulant on CM cheese	Pasteurization (65°C, 30 min), cooling (42°C), CaCl_2_, starter culture (incubation for 60 min), rennet coagulation (8 h)	CM cheese yield was improved by 14.6% by adjusting the fat content to 1.82%, total solid to 14%, and adding 1.5 mg of rennet powder to 100 ml of milk
Desouky et al. (55)	To evaluate the effect of concentration of CM powder (5–15%) on BM Cheddar cheese sauce quality	Cheddar cheese sauce was prepared by mixing hot water with disodium phosphate, sodium citrate, chopped cheese without or with CM powder replacement at 5, 10, and 15% ratio	The addition of increased ratio of CM powder in the blend improved the body and texture of the cheese sauce especially the ability to spread the sauce
Mbye et al. (11)	To evaluate the CM clotting activities of chymosin, citric, and acetic acid as compared to BM	Pasteurization (63°C, 30 min), cooling (40°C), addition of CaCl_2_ (3%), starter culture (3%), chymosin (50 IU/L), coagulation (8 h)	CM cheeses produced by camel chymosin and citric and acetic acid are much softer than those of BM
Bouazizi et al. (19)	Comparing the coagulation behavior of CM with that of cow's milk	Pasteurization (63°C, 30 min), cooling (35°C), addition of CaCl_2_ (0.02%), starter culture (3%), chymosin (55 IMCU/L), coagulation (2 h)	The composition, color, and texture were higher for cow cheese but panelists preferred CM cheeses
**Effect of processing conditions**			
Mehaia (56)	To determine the chemical composition, yield, and sensory characteristics of soft cheese prepared from CM by ultrafiltration	Pasteurization (65°C for 30 min), cooling (50°C), ultrafiltration (UF), cooling (42°C), addition of CaCl_2_ (0.02%) and starter culture (0.5%), 20 min), rennet (0.15/L), coagulation (3 h)	UF increased cheese yield, protein, fat and total solids recovery. CM cheese prepared by UF received a better sensory evaluation than conventional cheese
El Hatmi et al. (57)	A study to examine the impact of ultrafiltration (UF) and the addition of *Allium roseum* on CM cheese	Ultrafiltration, pasteurization (90°C, 10 min), cooling (45°C), addition of CaCl_2_ (0.2 g/L), starter culture (1%), camel chymosin, coagulation, addition of *A. roseum*	Cheese made using the UF process has a firmer texture, higher levels of protein, and a higher fat content. Moreover, cheese fortified with *A. roseum* had higher antioxidant activities
Kamal et al. (58)	To evaluate the rheological properties of rennet-induced coagulation of CM and BM under different pre-heating and salt addition	Pre-heating (50 & 70°C, 10 min), cooling (36°C, 5 min), enrichment with 10 or 20 mM CaCl_2_ or hydrogen phosphate dihydrate (Na_2_HPO4.2H_2_O), addition of rennet (6·25 μl in 25 ml milk)	In contrast to BM, preheating CM at 50°C negatively affected the gelation properties while preheating at 70°C prevented gel formation. Adding CaCl_2_ at 10 or 20 mM reduced the gelation time and increased gel firmness while adding Na_2_HPO_4_.2H_2_O at 10 or 20 mM induced the formation of weak gels from CM and BM pre-heated at 50°C and no gelation for CM pre-heated at 70°C
Konuspayeva et al. (59)	To evaluate the effect of calcium, lactation stage, and curd acidification on CM cheese quality	CM warming (20 or 36°C for 30 min), addition of calcium (CaCl_2_ at 0 or 50 g/L) or calcium phosphate powder), Chy-Max M (50 μL/L, strength 1,000 IMCU, 60 min), curd cut and filtered through cloth	No acceptable curd was obtained from CM before the 25–27 days of lactation. Less chymosin is required for the coagulation of raw than for pasteurized CM. The addition of calcium did not improve the CM curd in this case of heating at lower temperatures
Terefe et al. (60)	To identify the optimum conditions for coagulation of CM with partially purified *Moringa oleifera* enzyme extract	Pasteurization three temperature (55, 60, and 65°C), with three pH (4.5, 5, and 5.5), addition of 0.15 g/L CaCl_2_, addition of partially purified *Moringa oleifera* extract.at different volumes (0, 10, 20, 30, and 40%) in test tubes each containing 10 ml of milk, the clotting of the milk was observed	The highest camel milk clotting activity and curd firmness were observed at pH 5, temperature of 65°C and partially purified extract concentration of 10% for both seeds and leaves
Mbye et al. (61)	To evaluate the effect of pasteurization temperatures and high-pressure processing (HPP) on CM cheese quality	Pasteurization (65°C for 30 min, 75°C for 30 s) or HPP (350, 450, and 550 MPa for 5 min at 4°C), addition of CaCl_2_ (3%), starter culture (3%), chymosin (50 IMCU/L), coagulation (8 h)	Semi-hard CM cheeses were obtained after pasteurization (65°C, 30 min) or HPP (350 MPa, 5 min at 4°C) than treatments at high temperature or pressure
El Zubeir and Jabreel (62)	To evaluate the effect of the addition of different levels of NaCl on CM cheese quality	Camifloc cheese was made with different salt levels (0.0, 0.5, 1.0%)	CM cheese containing 1% salt had better sensory scores than 0.5% salt
Felfoul et al. (63)	To compare the effect of storage temperature (10 or 15°C) on physicochemical composition, texture, sensory, and structural properties of soft-brined camel and bovine cheeses obtained from skim milk and stored for up to 90 days for ripening	Pasteurization (63°C, 30 min), cooling (35°C), addition of CaCl_2_ (0.02%), starter culture (75 U/1L milk, camel chymosin (55 IMCU/L), coagulation (2 h) at 36°C. addition of 2% NaCl (wt/wt), stored at 10 and 15°C for 90 at controlled (80–90% Relative humidity	Camel and cow cheeses ripened at 15°C for 90 days had highest hardness values. However, CM cheese stored at 10°C was most appreciated by panelists due to over ripening at 15°C
**Effect of coagulants (acids, starter cultures, & enzymes)**			
Mehaia (64)	To define the manufacturing procedures for the production of fresh soft white cheese from camel milk, to determine its composition and yield, and to evaluate sensory properties of CM cheese produced by different methods	Three methods were used to prepare cheese from CM: (1) whole CM (15 L) containing salt (0, 1, 2, or 3%) or milk containing salt (3%) and different amounts of fat (0, 1, 2, or 3%) and rennet (0.004%, 2–3 h) and draining for 20–24 h, (2) whole CM (15 L) containing salt (3%), fat (0 or 1.5%), yogurt starter culture, and rennet, (3) lactic fermentation starter culture instead of yogurt starter culture. All cheeses were weighed, cut, packed in plastic bags, and stored at 5°C) for 1 day	Reduction of clotting time and improvement of renneting properties was achieved with reduction of pH, addition of calcium chloride prior to rennet addition (30 mg/100 g milk), increasing the amount of rennet (by 50–70 times). Use of yogurt starter (thermophilic) or lactic fermentation starter (mesophilic) culture increased the firmness of renin-coagulated cheeses
Khan et al. (65)	To compare cheese prepared from CM by acidification and starter culture plus chymosin	Pasteurization (65°C for 30 min), cooling (40°C), direct acidification (10% citric acid), or addition of starter culture (5%, 1 h) followed by rennet (0.15 ml/l), and coagulation (5 h)	Cheese prepared by starter culture and chymosin had a higher yield, total solids, protein, and fat than direct acidification
Benkerroum et al. (66)	To evaluate the effect of different levels of chymosin (Chy-Max) on CM cheese yield and microbiological quality	Pasteurization (71°C, 30 sec), cooling (37°C), addition of CaCl_2_ (0.02%), starter culture (3%, 90 min), chymosin (Chy-Max, 0.05–15 mL/L), and coagulation until a firm curd is visually observed	Chymosin at 1.7 mL/L gave better yield, and 2.9 mL/L of chymosin improved the sensory properties and microbiological quality
Ibrahim and Khalifa (67)	To examine the physicochemical and sensory properties of CM cheese treated with microbial transglutaminase (MTGase)	Pasteurization (72°C for 15 s), cooling 40°C, addition of salt (4%), starter culture (2%), CaCl_2_ (0.03%), rennet (1 ml/L), and 100 IU/L were added at the same time, then coagulation (7 h)	MTGase addition improved cheese yield, protein, total solids, and sensory attributes
Siddig et al. (68)	To investigate the effect of acid and starter culture on quality of white cheese from pure CM and a mixture of CM and bovine milk	Pasteurization (65°C, 30 min), cooling (40°C), addition of citric acid (10%) or starter culture, rennet (0.15 ml/L), and coagulation (5 h)	Cheeses made using starter cultures had higher protein, fat, and overall solids content than cheeses prepared using direct acidification
Wale et al. (69)	To evaluate the effect of the level of camel chymosin and cooking on coagulation properties and chemical composition, yield, texture, and sensory attributes of CM cheese	Pasteurization (65°C, 30 min), cooling (40°C), addition of CaCl_2_, starter culture (0.5%), camel chymosin (at 40, 70, or 100 IMCU/L), and cooking or no cooking of curd	Cooked cheese with 100 IMCU/L chymosin gave the highest values of protein, total solids, and hardness. However, the best overall sensory acceptance was for the cooked cheese made with 70 IMCU/L
Hailu et al. (70)	To compare the effects of levels of camel chymosin (55 and 85 IMCU L) and NaCl (2 and 5%, w/w) on CM cheese quality	Pasteurization (63°C, 30 min), addition of CaCl_2_ (0.02%), starter culture (75 U 1,000/L at 38°C), camel chymosin (55 or 85 IMCU/L), NaCl (2 or 5%), coagulation (2 h)	Harder cheese texture was obtained with 55 IMCU/L camel chymosin and 5% salt
Mihretie et al. (71)	To evaluate the coagulating effects of different levels of lemon juice on CM cheese	Tests were performed with different volumes of lemon juice (150, 200, 250, 300, 350, 400, 450, and 500 ml), coagulation (ambient temperature, 24 h)	Increased yield of cheese was observed in 500 ml of lemon juice added to 2L of CM. The cheese was fatty with high moisture content and had soft texture. The overall acceptability improved with the addition of lemon juice
Abou-Soliman et al. (72)	To evaluate how the level of (MTGase) after rennet addition impacts the properties of fresh CM cheese	Pasteurization (65°C, 30 min), cooling (40°C), addition of starter culture (0.2 g/L, 30 min), camel chymosin (30 min), MTGase (80, 100, or 120 U/L), and coagulation (3 h)	Soft CM cheeses with 80 U of MTGase added after 30 min of renneting has better yield, texture, and sensory properties
Fguiri et al. (73)	To assess the ability of enzyme extract from *Ficus carica* to coagulate CM	Pasteurization (65°C for 30 min), cooling (to 40°C), lowering pH (to 5.5), addition of starter culture and enzyme (1 mL/L), coagulation (37°C, 24 h)	1 mL of the enzyme extract in 100 mL of camel milk yields 15% CM cheese
Mbye et al. (11)	To evaluate the CM clotting activities of chymosin, citric, and acetic acid as compared to BM.	Pasteurization (63°C, 30 min), cooling (40°C), addition of CaCl_2_ (3%), starter culture (3%), chymosin (50 IU/L), coagulation (8 h)	CM cheeses produced by camel chymosin and citric and acetic acid are much softer than those of BM
Al-zoreky and Almathen (74)	To evaluate the effect of chymosin and (cultured or non-cultured) CM cheese	Pasteurization (63°C, 30 min), cooling (35°C), addition of CaCl_2_ (0.02%), starter culture (3%), chymosin (50 IMCU/L), coagulation (12 h)	CM cheese made from chymosin and tarter cultures had a higher cheese yield
Fguiri et al. (75)	To compare cheeses prepared by chymosin, ginger, pineapple, and kiwi extracts for their ability to clot CM in terms of yield and texture	Pasteurization (65°C, 30 min), cooling (40°C), addition of starter culture (*Lactococcus lactis*, 1 h), enzymes (10%), coagulation (37°C, 24 h)	Kiwi enzyme extract showed the highest potential for milk clotting of cheese, similar to camel chymosin
Mbye et al. (76)	To determine how *Withania coagulans*, camel chymosin, and mixtures of these enzymes clot CM	Pasteurization (65°C, 30 min), cooling (40°C), addition of 270 mmol CaCl_2_/L, starter culture (3%), chymosin (50 IMCU/L), coagulation (8 h)	A mixture of *Withania coagulans* and camel chymosin produced better quality cheese than either enzyme alone
Al-zoreky and Almathen (74)	To evaluate the coagulation of CM with recombinant camel chymosin with and without starter culture on cheese and whey properties	Pasteurization (63°C, 30 min), cooling (35°C), pH (6.2 adjusted with lactic acid), addition of calcium chloride (to 0.02% final concentration), YoFlex starter (DVS, at 0 or 0.05%, 37°C for 15 min), chymosin (CHY-Max M 2,500, 50 IMCU/L, 37°C, 110 min), refrigeration (24 h), addition of salt (1%)	Use of starter culture increased cheese yield (8.75%) and decreased the moisture content in the cheese and the leakage of fat and protein into the whey

Comparing cheeses prepared by mixing BM casein + BM whey, BM casein + CM whey, CM casein + BM whey, and CM casein + CM whey has shown that CM cheeses were smoother and less granulated than the BM cheeses (Figure 1). This effect is mainly due to differences in the casein fractions of the two types of milk (11), especially the very low proportion of κ-casein in CM (3.5% of the total caseins) compared to bovine (13%), sheep (9%), goat (20%), and buffalo (12%) milks (79). κ-casein is considered the primary factor responsible for coagulation of milk (17, 58, 80–82). However, the high level of β-casein in CM seems to also play an important role in this effect (11, 61, 76) in agreement to what has been described for BM (82). β-Casein possesses higher hydrophobicity than the other milk proteins and has more chaperone-like activities leading to suppression of protein aggregation (83). The high proportion of β-casein might be responsible for an “emulsifying effect” leading to a smooth texture and increased water retention in CM cheeses (11).

A study on cheese made from camel caseins plus either CM or BM whey proteins showed that the addition of BM whey provided slightly harder cheeses compared to the addition of CM whey suggesting a role for whey proteins (11). The differences between CM and BM whey proteins (Table 2) and the amounts and types of associated fats and calcium may also contribute to cheese curd gelation time and cheese quality (84). β-Lactoglobulin variant B was found to cause higher yield of BM cheese on dry weight basis compared to the A or AB variants at all levels of addition (0–1.35%) (85). However, the effect of whey proteins on cheese quality will depend on concentration. Whey proteins were suggested to associate with the casein micelle gels leading to higher cheese yield but this association reaches a saturation point at 0.675% whey protein addition possibly due to steric hindrance by inhibiting the access of rennet to the casein micelles during the primary stage of coagulation or by inhibiting the aggregation of the micelles during the second stage. This might explain the different results reported on the effect of BM whey proteins on cheese yield and firmness (86–93). The contribution of CM whey proteins to cheese quality requires further investigations.

**Figure 2:**
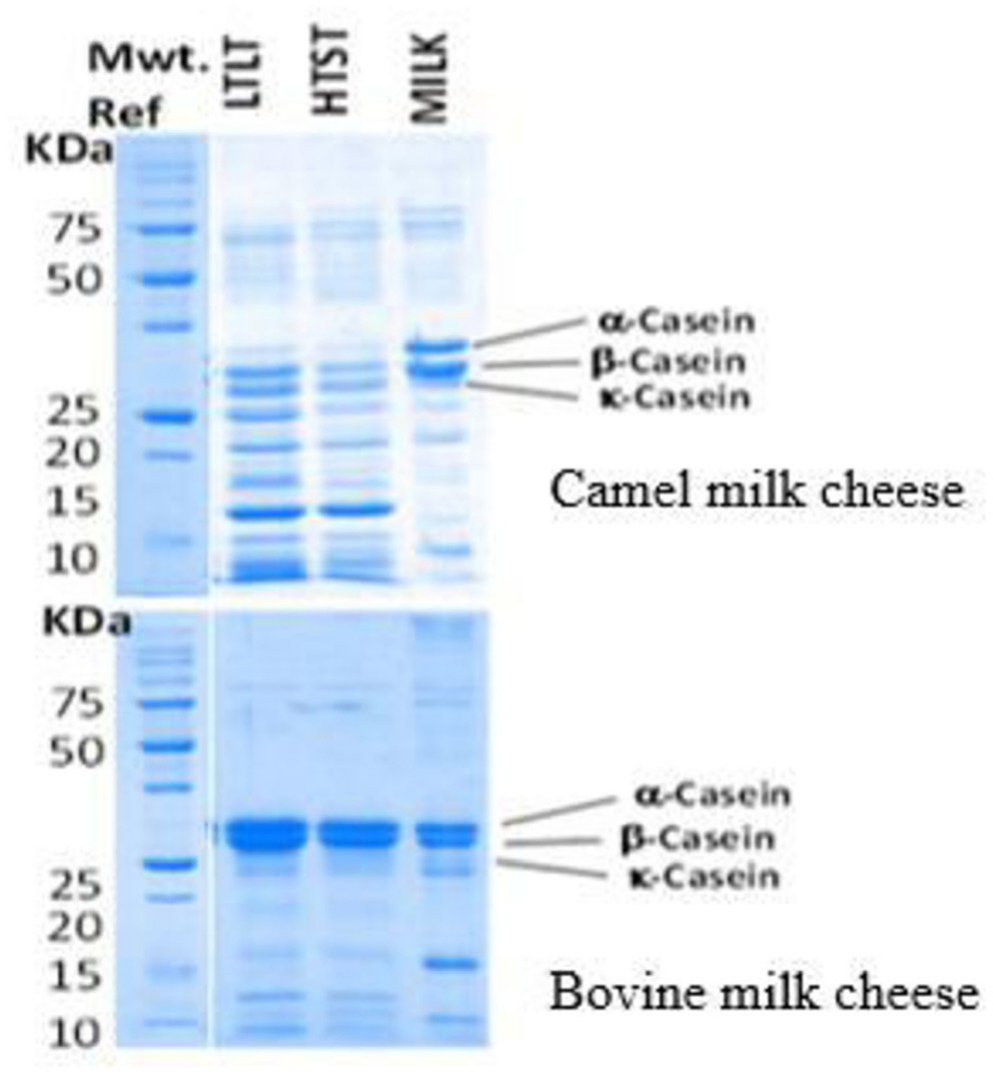
SDS-PAGE electropherograms of the camel and bovine milk cheeses showing proteolysis in the camel milk cheeses.

**Figure 3:**
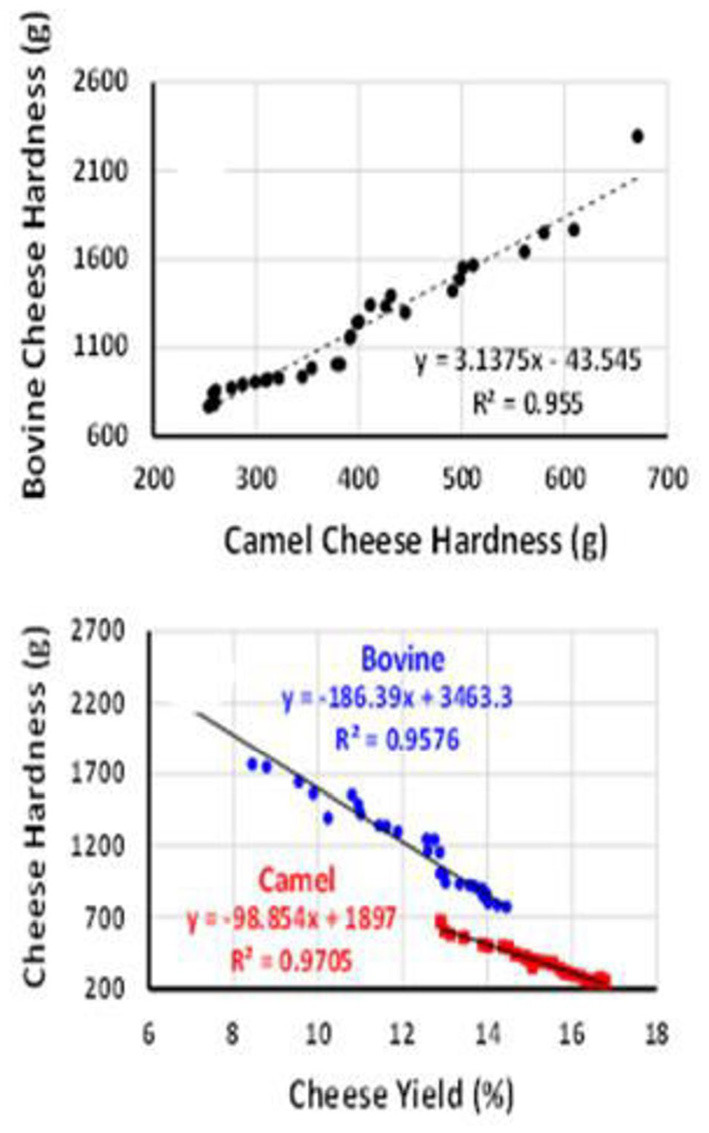
Correlations between camel and bovine cheese hardness and yield showing similar trends but different magnitudes. Source: Mbye et al. (76).

SDS-PAGE electrophoresis shows clear differences in the protein and peptide profiles of CM and BM cheeses and whey (Figure 2). The CM cheeses show several low molecular weight bands suggesting that excessive proteolysis of caseins has occurred in these cheeses (61, 76). Endogenous enzymes such as plasmin in CM (94–96) might be responsible for the hydrolysis of caseins, especially β-casein. The peptides produced by the proposed hydrolysis can contribute to the fine and smooth texture and water retention causing a soft texture of CM cheeses (82). Some of the low molecular weight peptides from CM cheese seem to migrate into the whey fraction, explaining the low total solid content in CM cheeses and the casein bands observed in the SDS-PAGE results of whey. In addition, fresh CM coagula were found to retain more moisture than those of BM representing yet another factor that contributes to the softness and high yield of fresh cheese (Figure 3).

**Figure 4:**
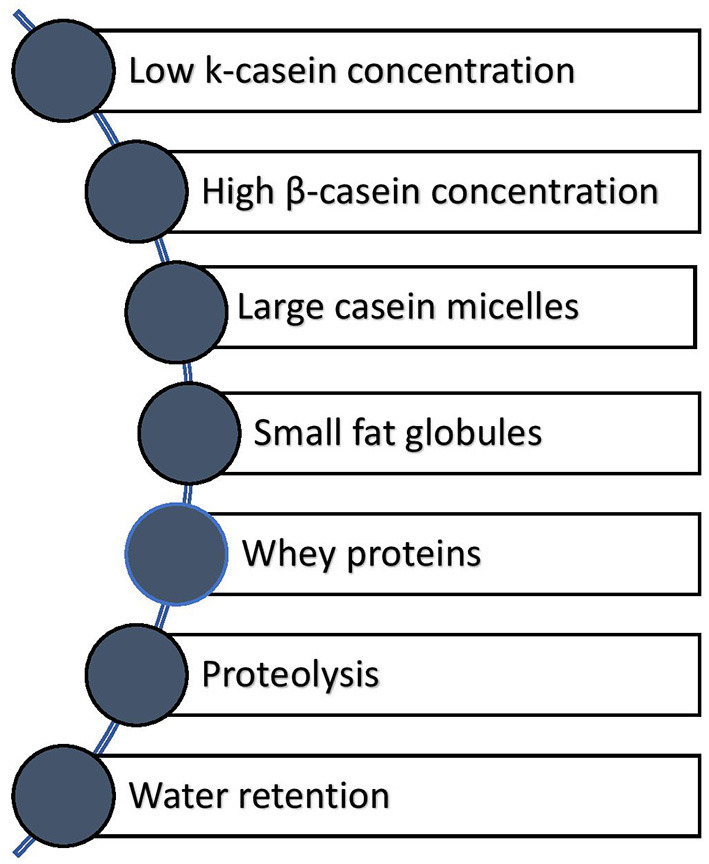
Milk compositional factors that contribute to the soft texture of CM cheese.

Figure 4 illustrates the factors that all contribute collectively to the soft texture of CM cheeses. The effect of these factors on CM cheese texture needs to be studied further.

## Effects of Processing Conditions

It is important to note that the processing conditions for manufacturing CM cheeses significantly impact their yield, nutritive value, safety, and sensory quality (97). For example, milk pasteurization temperature, high-pressure treatment, calcium chloride content, and pre-acidification substantially affect the final cheese hardness (Table 3). For example, increasing the total solid content in CM by ultrafiltration was found to improve the cheese yield, and protein and fat recovery rates by 45, 40, and 42%, as well as improving the sensory properties of cheese compared to conventional processing (56, 57).

Milk pasteurization is vital to assure CM cheese's safety (98). However, high pasteurization temperature could affect the quality of cheese, such as yield and texture (61, 99, 100). High temperatures are known to enhance the formation of κ-casein complexes through disulfide bonds between whey proteins and casein micelles, which may hinder casein coagulation (101). In addition, high temperature leads to undesirable changes in sensory attributes and nutritional value of the product (102). It is recommended to avoid pasteurizing cheese milk for more than 65°C for 30 min or 72°C for 15 s (27, 48). It was found that CM coagulates slowly when the temperature exceeds 65°C for 30 min resulting in cheeses with weak gels (18, 99, 103, 104).

Studies have shown that the coagulation properties (coagulation time, rate of curd firming) as well as the yield) improved after HPP treatment of camel milk (61). This is also the case for bovine milk (105, 106) and caprine milk (107). HPP treatment at 200–400 MPa has been reported to enhance CM coagulation and coagulum strength (61, 108) but HPP treatment at 600 and 800 MPa inhibited clotting (108). Due to its ability to inactivate gram-positive and gram-negative bacteria at room temperature, high-pressure processing (HPP) is used in the food industry as a preservation technique (109). HPP treatments above 400 MPa affects the conformational structure of the casein micelles by weakening their electrostatic and hydrophobic interactions leading to dis-aggregation of micellar fragments and enhancement of milk physiochemical properties and technological applications (110, 111). The disruption of casein micelles leads to an increased surface area, causing faster rennet coagulation (112). High pressure of 500 MPa denatures β-lactoglobulin leaving the immunoglobulin and α-lactalbumin intact (113) and also causes modification of the fat globules (109).

In addition to protein and fat, calcium is known to play an essential role in improving the gelation, yield, and hardness of BM cheeses (114–118). Calcium enhances the interactions within and between the casein micelles by shrinking and stabilizing the porous network inside (115, 119, 120). The addition of calcium salts to skim BM decreases the pH (114) and this helps to reduce the milk coagulation time (116) and to improve rennet coagulation (115). Results on the effect of calcium addition on CM cheese quality has not been conclusive. While some studies showed that adding calcium chloride before rennet reduces the coagulation time and improves CM cheese yield (11, 56, 62, 65, 121), other studies showed no effect (59, 121). One study has concluded that the effect of calcium is pH-dependent (6.6–6.0) and that 0.02% calcium at pH 6.3 reduced coagulation time (122). It is also possible that the effect of calcium is affected by prior CM heating, e.g., in contrast to pasteurized milk, the addition of calcium did not affect the coagulation and quality of cheese made from CM heated at 20 or 36°C for 30 min (59).

Pre-acidification prior to enzyme addition is a necessary step in the manufacture, ripening, and quality of many cheese types (123, 124). Pre-acidification enhances nutrient contents and improves the texture, flavor, and other organoleptic characteristics, inhibits microbial spoilage and enhances coagulant activity and retention in cheese curds. Milk acidification could be done directly, by adding acid or glucono-6-lactone, or more commonly indirectly *via* the use of cultures able to produce lactic acid. Thus, making CM cheese requires the acidification to lower the pH to around 6.4 before adding enzymes to decrease the clotting time by 28% (125). Some studies reported that reducing the pH of CM to 5.6 at temperatures up to 42°C further reduces the coagulation time (126, 127).

## Effect of Coagulants

Coagulants used in cheese preparation include animal rennins such as pepsin and chymosin, plant-based proteases, starter cultures, or organic acids for acidification. Milk coagulation with proteolytic enzymes proceeds by destabilization and precipitation of the casein micelles due to hydrolysis by enzymes or precipitation by acids after neutralizing the negative charges of k-casein (11, 19, 57, 70, 74, 122, 128–130).

The rennet enzymes are aspartic peptidase, and the most used are the combinations of chymosin A, B, C, and pepsin extracted from the stomach of calves and other ruminants (131). Renins disrupt the milk emulsion and separate caseins from the whey, causing them to coagulate into cheese by cleaving κ-casein into para-κ-casein and casein macropeptide (132, 133). Many studies have consistently found that the coagulation of CM proceeds at much lower rates and produces a more fragile coagulum compared to BM when using bovine chymosin (125, 134–137). It was, however, shown that camel chymosin has 70% more clotting activity for BM compared to bovine chymosin, which does not efficiently coagulate CM (121). It has also been shown that camel chymosin from older camels was the most effective milk clotting agent in camel and bovine milk (128). The shortage of coagulation enzymes prompted the industry to look for alternative proteolytic enzymes with similar action (138), which has led to the production of several microbial recombinant chymosin products as substitute for animal rennet in cheese manufacturing (139). The high clotting activity of camel chymosin makes it an attractive option for small and large-scale cheese production (140, 141). However, recombinant enzymes are unpopular in some countries due to religious matters and diets (142).

Camel and bovine chymosin selectively cleave the Phe97-Ile98 bond in camel κ-casein compared to the Phe105-Met106 bond in bovine κ-casein. This causes the hydrophilic C-terminal to dissociate from κ-casein leading to the destabilization of the casein micelles and resultant aggregation and coagulation of the milk. The improved milk-clotting activity of camel chymosin has been attributed to better substrate binding, which is facilitated by its surface charge (143). Recombinant camel chymosin is produced by the expression of the camel chymosin gene in a strain of *Aspergillus niger* (121, 143). Recent studies have evaluated the use of camel chymosin to make soft white cheese from CM and found that combination of chymosin and thermophilic starter culture increases the cheese yield (74, 129).

In the past few years, the challenge associated with cheese yield and quality from CM has also contributed to exploring alternative rennet enzymes from plant origin (144–146). Plant proteases have been divided into groups based on the hydrolytic process mechanism: aspartate, serine, and cysteine proteases. Serine protease such as *Zingiber officinale* extracts (147), cysteine proteases isolated from *Ficus carica* (73), and aspartic proteases from *Withania coagulans* (76) have been used in CM cheese production and the resultant cheeses were found acceptable. The effects of camel chymosin and *Withania coagulans* extracts on the coagulation of CM and BM cheeses' yield and textural quality have been compared and again, CM was found to have a longer gelation time and softer cheese compared to BM (76). This study showed that the yield of un-ripened CM cheese produced by chymosin or the *Withania* extracts was consistently higher than that of BM cheeses due to higher moisture entrapment, which led to reduced cheese hardness (Figure 3). This study also showed that optimal CM as well as BM cheese hardness was obtained by clotting the milks with mixtures of *Withania* extracts and chymosin suggesting some synergistic interactions, an effect that deserves further investigations.

Cheese from CM can also be obtained by direct acidification with lemon juice or organic acids. The acid coagulation affects the stability of casein micelles by neutralizing their negative charges and destabilizing the micelles by dissolving some colloidal calcium phosphate crosslinks and altering internal bonding between proteins. The development of aggregates and eventually gelation occurs at the isoelectric point when electrostatic repulsion is insufficient to overcome attractive forces (148, 149). The manufacturing of CM cheeses using acetic acid has been documented by Mohamed et al. (150) and Mbye et al. (11) while Mihretie et al. (71) made CM cheese using acids from citrus fruits and the result showed that CM could be coagulated by citric acid. CM cheeses prepared using organic acids were found to have higher moisture than those produced by chymosin suggesting that the enzymatic hydrolysis of k-casein by chymosin modifies the gels' structure make them more porous, which lead to lower water retention and firmer gel.

Among the most recent studies looking at the quality of CM soft cheese, the effect of microbial transglutaminase (MTGase) on CM cheese quality has been evaluated (67, 72). MTGase enhanced the properties of soft cheese and an excellent sensory quality score was dependent on the concentration and timing of addition, e.g., adding 80 units of MTGase to milk after renneting produced the highest solids and protein contents. Only few published studies have allowed ripening of CM cheese in salt or brine solutions or in the cheese whey (53, 63, 64).

## Summary and Conclusion

**Figure 5:**
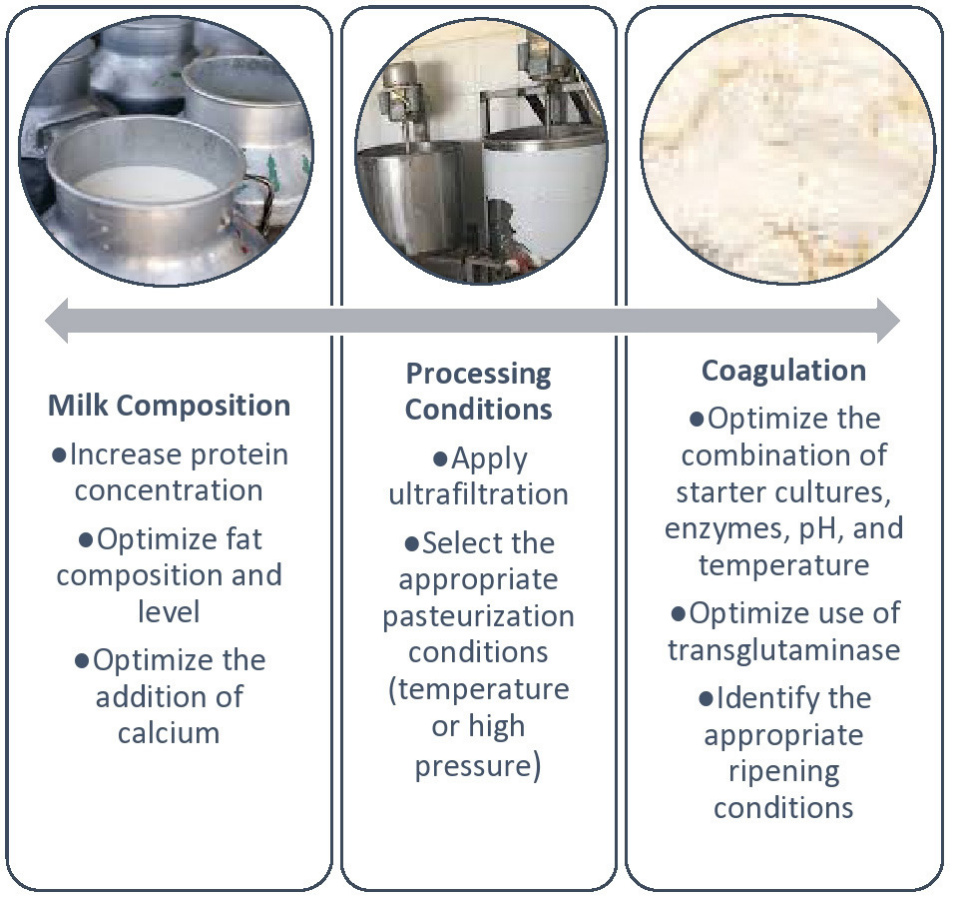
Important considerations during the preparation of camel milk cheese.

Figure 5 summarizes the important compositional, processing, and coagulation factors that need to be considered during the preparation of cheese from CM. CM cheese production has significantly changed due to availability of camel chymosin. The softness of CM cheeses has been mainly attributed to the low level of k-casein in the milk and larger casein micelles size. However, other factors seem to contribute to this effect as shown in Figure 4. Endogenous milk proteases, e.g., plasmin and cathepsinthe, may decompose the caseins and produce high and low-molecular-weight peptides. Proteolysis can also occur as a result of the action of residual rennet or other coagulants retained in the curd after milk coagulation and by enzyme action of both the starter cultures and non-starter cultures. CM cheese with a strong curd and higher solid content had a more appealing sensory profile than CM cheese with a weak curd. Milk pasteurization at temperatures not exceeding 65°C for 30 min or high-pressure processing are more effective in providing cheeses with a firm texture. In general, CM is better suited for producing soft cheeses that are also liked by the consumers in sensory evaluation studies. To better understand the effect of different milk proteins and processing conditions on the texture and quality of CM cheese, further studies are needed. Furthermore, shelf-life studies should be conducted to determine how storage conditions affect the quality of CM cheese.

## Author Contributions

AK-E was in charge of conceptualizing the ideas and funding and. MM wrote the manuscript draft. All authors reviewed and contributed to the final draft. All authors contributed to the article and approved the submitted version.

## Funding

This research was funded by research grant 31F133 from United Arab Emirates University.

## Conflict of Interest

The authors declare that the research was conducted in the absence of any commercial or financial relationships that could be construed as a potential conflict of interest.

## Publisher's Note

All claims expressed in this article are solely those of the authors and do not necessarily represent those of their affiliated organizations, or those of the publisher, the editors and the reviewers. Any product that may be evaluated in this article, or claim that may be made by its manufacturer, is not guaranteed or endorsed by the publisher.
